# Purification, Structural Characterization, and Immunomodulatory Activity of an Exopolysaccharide from *Acetilactobacillus jinshanensis* BJ01 in *Baijiu* Fermentation Grains

**DOI:** 10.3390/foods14132162

**Published:** 2025-06-20

**Authors:** Tian Tian, Bo Wan, Ying Xiong, Han Wang, Yuanyuan An, Ruijie Gao, Pulin Liu, Mingchun Zhang, Lihong Miao, Weifang Liao

**Affiliations:** 1 School of Life Science and Technology, Wuhan Polytechnic University, Wuhan 430023, China; t15137029987@163.com (T.T.); bo.wan@foxmail.com (B.W.); 17373604579@163.com (Y.X.); n2848825099@163.com (Y.A.); rjgao1989@whpu.edu.cn (R.G.); plliu@whpu.edu.cn (P.L.); 2 Hubei Institute of Common Steroid Drugs Co., Ltd., Wuhan 430200, China; 17702747819@163.com; 3 Hubei Baiyunbian Liquor Industry Co., Ltd., Songzi 434200, China; zmc700196@163.com

**Keywords:** *Acetilactobacillus jinshanensis* BJ01, exopolysaccharide, structural characterization, immunomodulatory activity

## Abstract

This study aims to identify the chemical structure and immunomodulatory activity of exopolysaccharides (EPSs) from *Acetilactobacillus jinshanensis* BJ01 and suggest its potential applications in the pharmaceutical field and as functional food additives. The EPS-1 produced by *A*. *jinshanensis* BJ01 was purified using column chromatography. The lyophilized powder obtained by vacuum freeze-drying was used for structural characterization and immunomodulatory activity analysis. Gel permeation chromatography (GPC) determined its molecular weight as 156.58 kDa. High-performance anion-exchange chromatography (HPAEC) identified that the EPS-1 is composed of mannose, xylose, and glucose. The structural characterization of EPS-1 by gas chromatography–mass spectrometry (GC-MS) and 1D/2D nuclear magnetic resonance (NMR) spectroscopy demonstrated that EPS-1 is primarily composed of α-D-Man*p*-(1→, →2,6)-α-D-Man*p*-(1→, →2)-α-D-Man*p*-(1→, and →3)-α-D-Man*p*-(1→. Scanning electron microscopy (SEM) and atomic force microscopy (AFM) illustrated that EPS-1 exhibited a round, flake-like morphology. In vitro experiments with RAW264.7 macrophages demonstrated the high immunomodulatory activity of EPS-1. Significant upregulation of iNOS, IL-6, and TNF-α mRNA levels was confirmed by qRT-PCR (*p* < 0.05). Western blotting revealed that EPS-1 (6.25 μg/mL) induced phosphorylation of NF-κB (p65, IκBα) and MAPK (ERK) signaling proteins. This study provides the first structural and immunomodulatory characterization of an exopolysaccharide from *A. jinshanensis* BJ01, highlighting its potential as a novel immune adjuvant.

## 1. Introduction

Microbial exopolysaccharides (EPSs) are high-molecular-weight polymers synthesized and secreted by microorganisms into the extracellular environment, consisting of multiple monosaccharides linked by glycosidic bonds to form linear or branched structures [1,2]. In recent years, EPSs produced by microorganisms have attracted significant research interest due to their stable properties, low production costs, ease of separation, and safety as non-toxic compounds [3,4].

The structure of microbial EPSs is remarkably varied and intricate. Typically, the investigation of EPS structures includes the composition and arrangement of monosaccharides, the range of molecular weights, types of glycosidic bonds, repeating units, linkage sites, and overall spatial configuration [5]. Their molecular weight typically ranges from 10^4^ to 10^6^ Da [6]. In terms of monosaccharide composition, microbial EPSs commonly contain glucose, galactose, mannose, xylose, and fucose [7]. FT-IR spectroscopy is frequently employed to analyze and identify the various functional groups present in polysaccharides [8]. Methylation analysis identifies the types and proportions of monosaccharides, clarifying the linkage sites and positions of glycosidic bonds. NMR spectroscopy provides additional characterization by detailing the types of monosaccharide residues, their sequences, positions, and the configurations of glycosidic bonds [9]. The higher-order structures of polysaccharides are often analyzed using techniques such as SEM, AFM, light scattering, and computer-aided techniques to observe morphological characteristics, detect polymorphic features, and construct complex structural models [10]. Since structure determines function, different structural configurations impart unique physical and chemical properties to EPS, such as solubility, viscosity, and gelling ability, while also conferring various biological activities [11,12,13]. Thus, structural analysis is crucial for comprehending the biological activities of EPSs. However, elucidating the precise relationship between the structure and function of EPSs remains a significant challenge in current research.

Current research demonstrates that EPSs possess a wide range of potential applications. In the medical field, EPSs exhibit beneficial biological activities, including anti-tumor, antiviral, and antioxidant properties, as well as promoting immune modulation and regulating the digestive system [14]. A study found that EPSs produced by the marine-derived probiotic *Pediococcus pentosaceus* may inhibit colon cancer (Caco-2) and breast cancer (MCF-7) cell lines by inducing apoptosis and competing with growth promoters at cell receptors [15]. Another study demonstrated that EPSs produced by *Lactobacillus delbrueckii* TUA4408L enhanced resistance to rotavirus by reducing viral replication and modulating the inflammatory response [16]. Regarding antioxidant activity, researchers found that EPSs from the *Leuconostoc mesenteroides* 187 strain, isolated from Sichuan pickles, exhibited strong in vitro antioxidant properties [17]. Additionally, the polysaccharide extracted and purified from *Aureobasidium pullulans* CGMCC 23063 has demonstrated immunomodulatory activity in RAW 264.7 cells [18]. EPSs have been shown to modulate intestinal microbiota and promote digestive well-being [19]. In the food industry, some microbial EPSs contribute to the unique texture and flavor of fermented dairy products, serve as safe food additives, and are potential sources of food-grade polysaccharides. They are widely used as thickeners, stabilizers, emulsifiers, gelling agents, and for water retention [20]. Microbial extracellular polysaccharides also offer unique advantages, including renewability, sustainable production, excellent biocompatibility, and ease of modification through genetic and metabolic engineering [21,22]. Currently, the application of EPSs is limited by low yields and inconsistent quality. Therefore, developing new EPS resources from lactic acid bacteria is of significant importance.

*Acetilactobacillus jinshanensis* BJ01 is a Gram-positive bacterium originally isolated from the traditional Chinese *Baijiu* fermentation process. As a representative strain of the newly established genus *Acetilactobacillus*, it holds significant research value [23]. This research focused on the effective extraction and refinement of the EPS-1 from *A. jinshanensis* BJ01, utilizing DEAE-Sepharose Fast Flow column chromatography and Sepharose CL-6B molecular sieving techniques, resulting in the characterization of a new polysaccharide. The primary structure of EPS-1 was determined through chemical analysis, and its morphological characteristics were observed using SEM and AFM. Additionally, the immunomodulatory activity of EPS-1 was evaluated through in vitro experiments using RAW 264.7 macrophages. The effects of EPS-1 on cytokine expression were assessed using ELISA and qRT-PCR, and the potential signaling pathways involved were analyzed by Western blot.

## 2. Materials and Methods

### 2.1. Materials and Reagents

The subject of this study is an acid-tolerant lactic acid bacterium, *Acetilactobacillus jinshanensis* BJ01 CCTCC M2021419, which was isolated from *Baijiu* fermentation grains. The DEAE-Sepharose Fast Flow and Sephadex CL-6B resins were acquired from Beijing Solarbio Science & Technology Co., Ltd. (Beijing, China). Lipopolysaccharide (LPS) derived from *Escherichia coli*, 0.1% neutral red solution, and monosaccharide standards (sucrose, glucose, mannose, galactose, ribose, arabinose, and galacturonic acid) were all purchased from Sigma Chemical Co., (St. Louis, MO, USA). Fetal bovine serum (FBS), phosphate-buffered saline (PBS), penicillin/streptomycin, MTT assay kit (3-(4,5-dimethylthiazol-2-yl)-2,5-diphenyltetrazolium bromide), and Dulbecco’s Modified Eagle Medium (DMEM) were obtained from Thermo Fisher Scientific Inc. (Waltham, MA, USA). Nitric oxide (NO) assay kits were acquired from Beijing Boxbio Science & Technology Co., Ltd. (Beijing, China), and ELISA kits for IL-6 and TNF-α were purchased from Jiangsu Meimian Industrial Co., Ltd. (Yancheng, China). All other reagents used in this study were of analytical grade and obtained from Shanghai Aladdin Biochemical Technology Co., Ltd. (Shanghai, China), and Shanghai Macklin Biochemical Co., Ltd. (Shanghai, China), and the MRS culture medium for microbial cultivation was sourced from the Oxoid product line of Thermo Fisher Specialty Diagnostics Ltd. (Waltham, MA, USA).

### 2.2. Extraction and Purification of EPS

Before culturing *A. jinshanensis* BJ01, an acidic *Man–Rogosa–Sharpe* (MRS) liquid medium was prepared with the following components per liter: 10 g of beef extract, 10 g of peptone, 5 g of yeast extract, 20 g of glucose, 5 g of anhydrous sodium acetate, 2 g of dipotassium hydrogen phosphate, 2 g of ammonium citrate, 1 mL of Tween 80, 0.2 g of magnesium sulfate heptahydrate, and 0.05 g of manganese sulfate monohydrate, dissolved in 1000 mL of deionized water. The pH was adjusted to 3.8 using a mixed acid solution (lactic acid–acetic acid = 1:1, *v*/*v*), followed by sterilization via autoclaving. *A. jinshanensis* BJ01 was then inoculated into the acidic MRS medium at a 5% inoculation ratio and incubated statically at 30 °C for 120 h [23].

After fermentation, the broth was heated in a 60 °C water bath for 6 h to achieve the desired state for subsequent separation, followed by centrifugation at 8000 rpm for 20 min to collect the supernatant. To remove proteins, 80% (*m*/*v*) trichloroacetic acid (TCA) was added to the supernatant to a final concentration of 5%, followed by incubation at 4 °C overnight. Following protein precipitation, the mixture was centrifuged at 8000 rpm for 20 min. Three volumes of 95% ethanol were added to the fermentation broth, and the mixture was left to stand at 4 °C for 24 h. The flocculent polysaccharide precipitate was then collected by centrifugation at 8000 rpm for 20 min. The precipitate was subsequently dissolved in ddH_2_O and dialyzed against distilled deionized water using a dialysis membrane with an 8–14 kDa molecular weight cut-off for 48 h at 4 °C, with dialysis water refreshed every 8 h. Finally, the EPS was lyophilized in a vacuum freeze-dryer for 24 h to obtain a dry powder. The polysaccharide yield was quantified using the phenol–sulfuric acid method [24].

A total of 0.1 g of crude polysaccharide was dissolved in 5 mL of 55 mmol/L Tris-HCl buffer and filtered through a 0.45 μm membrane. Initial purification was carried out using a DEAE-Sepharose Fast Flow anion-exchange chromatography column [25], with stepwise elution using Tris-HCl buffer containing 0.0, 0.1, 0.3, 0.5, and 0.7 mol/L NaCl. The partially purified polysaccharide fractions were dialyzed against deionized water for 48 h and lyophilized. Subsequently, 0.1 g of the preliminarily purified polysaccharide was redissolved in 5 mL of 55 mmol/L Tris-HCl buffer, filtered through a 0.45 μm membrane, and further purified using a Sepharose CL-6B gel filtration column [26]. The final eluted polysaccharide solution was dialyzed for 48 h in deionized water and then freeze-dried under vacuum to obtain the purified EPS-1.

### 2.3. EPS-1 Characterization

#### 2.3.1. UV-vis Spectroscopy

The UV-vis absorption spectrum of EPS-1 was recorded using a UV-vis spectrophotometer (Shimadzu UV-2600, Kyoto, Japan). The lyophilized EPS-1 sample was dissolved in ultrapure water at a concentration of 0.5 mg/mL and scanned over a wavelength range of 200–800 nm at room temperature. Ultrapure water was used as the blank control. The spectrum was used to assess the presence of proteins or nucleic acids based on absorbance at 260 nm and 280 nm.

#### 2.3.2. Determination of Moisture Content, Solubility, Viscosity, and Gelling Capacity of EPS-1

Moisture content was determined by placing 0.5 g of lyophilized EPS-1 powder into a moisture analyzer (OHAUS Instruments, Changzhou, China) and drying at a constant temperature of 105 °C until a stable weight was achieved. To determine solubility, 30.0 mg of EPS-1 was dissolved in 1.0 mL of deionized water, dispersed thoroughly using magnetic stirring at 35 °C for 30 min, and then centrifuged at 8000× *g* for 15 min. The supernatant was collected and dried at 80 °C to a constant weight. The solubility of EPS-1 was calculated based on the mass of the dried residue relative to the original sample mass [27]. The apparent viscosity of a 1.0% (*w*/*v*) EPS-1 aqueous solution was measured at 25 °C using a modular rheometer (Anton Paar MCR 502 TwinDrive-ready). The shear rate range was set from 0 to 100 s^−1^. The gelling capacity of EPS-1 was preliminarily evaluated using the test tube inversion method. A 1.0% (*w*/*v*) solution of EPS-1 was heated in a water bath at 95 °C for 30 min, cooled at room temperature for 30 min, and subsequently stored at 4 °C for 24 h. Gel formation was assessed based on the flow behavior of the sample upon tube inversion [28].

#### 2.3.3. Determination of Molecular Weight

The homogeneity and molecular weight of various fractions were measured using SEC-MALLS-RI. The EPS-1 was dissolved in 0.1M NaNO_3_ aqueous solution containing 0.02% NaN_3_ at the concentration of 1 mg/mL and filtered through a filter of 0.45 μm pore size. The weight and number-average molecular weight (Mw and Mn) and polydispersity index (Mw/Mn) were measured on a DAWN HELEOS-II laser photometer (Wyatt Technology Co., Goleta, CA, USA), equipped with two tandem columns (300 × 8 mm, Shodex OH-pak SB-805 and 803; Showa Denko K.K., Tokyo, Japan). The flow rate was maintained at 0.6 mL/min, with the column temperature regulated at 45 °C. A differential refractive index detector (Optilab T-rEX, Wyatt Technology Co., USA) was simultaneously connected to give the concentration of fractions and the dn/dc value.

#### 2.3.4. Analysis of Monosaccharide Composition

The analysis of monosaccharide composition was conducted based on a method established in previous research, incorporating minor adjustments to improve its applicability [29]. Hydrolysis of a 5 mg sample of EPS-1 was carried out using 2 M TFA in a sealed tube at 121 °C for a period of 2 h. Subsequent to this process, the EPS-1 was dehydrated under a nitrogen atmosphere. The residue was then washed with methanol and dried under nitrogen, repeating the methanol wash two to three times. Following drying, the residue was rehydrated in deionized water and subsequently filtered using a 0.22 μm microporous membrane for further examination. The assessment of EPS-1 was carried out using high-performance anion-exchange chromatography (HPAEC) with a CarboPac PA-20 anion-exchange column (3 × 150 mm; Dionex, Singapore) and a pulsed amperometric detector (PAD; Dionex ICS 5000+ system). The flow rate was set at 0.5 mL/min, with an injection volume of 5 μL. The solvent system comprised three components: solvent A (ddH_2_O), solvent B (0.1 M NaOH), and solvent C (0.1 M NaOH, 0.2 M NaAc). The gradient elution commenced with a volume ratio of A:B as 95:5:0 at 0 min, shifting to 85:5:10 at 26 min, and this ratio was maintained until 42 min. At 42.1 min, the composition changed to 60:0:40, followed by 60:40:0 at 52 min, ultimately reverting to 95:5:0 at 52.1 min, with this ratio held until the end of the run at 60 min.

#### 2.3.5. FT-IR Analysis

The FT-IR spectra of the EPS-1 was acquired using a Nicolet iZ-10 spectrometer (Thermo Nicolet, USA). EPS-1 was combined with KBr powder and compressed into pellets with a thickness of 1 mm. The FT-IR measurements were conducted over a spectral range of 4000 to 400 cm^−1^.

#### 2.3.6. Methylation Analysis

The methylation of EPS-1 was performed according to a previously established protocol with slight modifications [30]. The EPS-1 was dissolved in dimethyl sulfoxide (DMSO) and subjected to methylation in a DMSO–NaOH solution using methyl iodide (CH_3_I). Following the completion of the methylation process, the permethylated derivatives were hydrolyzed using 2 M TFA at 121 °C for 1.5 h. Sodium borodeuteride (NaBD_4_) was used to reduce the hydrolysate, which was then acetylated in acetic anhydride at 100 °C for 2.5 h. The resulting acetylated compounds were dissolved in chloroform and subjected to analysis by GC-MS employing an Agilent 6890A-5975C system, fitted with an Agilent BPX70 column (30 m × 0.25 mm × 0.25 µm; SGE Analytical Science, Trajan Scientific and Medical, Victoria, Australia). High-purity helium was employed as the carrier gas, utilizing a split ratio of 10:1 and an injection volume of 1 μL. The mass spectrometry analysis commenced at the initial temperature of 140 °C for 2 min, then ramped up to 230 °C at a rate of 3 °C/min for 3 min, with data acquisition in SCAN mode covering a mass-to-charge ratio (*m*/*z*) range from 50 to 350.

#### 2.3.7. NMR Analysis

EPS-1 was prepared by dissolving it in 0.5 mL of deuterium oxide (D_2_O) to achieve a concentration of 40 mg/mL. Next, 1D-NMR and 2D-NMR spectra, including proton NMR (^1^H-NMR), carbon NMR (^13^C-NMR), HSQC, and COSY, were obtained at 25 °C using a Bruker AVANCE NEO 500 MHz spectrometer (Bruker, Rheinstetten, Germany). The liquid probe QXI ^1^H/^31^P/^13^C/^15^N 5 mm four-channel inverse detection probe (z-gradient and ATM Acc) has the following technical specifications: signal-to-noise ratio (^1^H): 888; resolution (Hz): 0.32 (rotating). The observation probe BBFO ^1^H-^19^F, ^31^P-^15^N, ¹H decoupling/observe multinuclear forward detection probe (z-gradient and ATM) has the following technical specifications: signal-to-noise ratio (^1^H): 798; resolution (Hz): 0.26 (rotating); signal-to-noise ratio (^13^C): 328; resolution (Hz): 0.1.

#### 2.3.8. SEM and AFM Observation

In this investigation, the microstructural characteristics of EPS-1 were examined using SEM and AFM [18]. The morphological features of the polysaccharides were examined using a Zeiss Merlin Compact scanning electron microscope (Jena, Germany). EPS-1 was coated with a thin layer of gold and placed onto a substrate, with imaging performed at a voltage of 3.0 kV under high vacuum, utilizing magnifications of 10 K and 5 K. For AFM analysis, EPS-1 was initially dissolved in distilled water at a concentration of 1 mg/mL and then gradually diluted to 10 μg/mL before being applied to substrates for observation.

### 2.4. In Vitro Immunomodulatory Activity of EPS-1

#### 2.4.1. Cell Culture

The RAW 264.7 murine macrophage cell line was cultivated in DMEM enriched with 10% FBS and 1% penicillin–streptomycin (100 U/mL). Incubation occurred at 37 °C in a humidified chamber containing 5% CO_2_, with subculturing executed in accordance with the observed growth parameters.

#### 2.4.2. Assessment of Cell Proliferation

RAW 264.7 macrophage proliferation was assessed using the MTT assay, with minor modifications to a previously described method [31]. Briefly, RAW 264.7 cells were initially seeded in 96-well plates at a concentration of 1 × 10^5^ cells/mL and incubated for 24 h. Subsequently, 100 μL of EPS-1 at various concentrations (0, 5, 6.25, 12.5, 25, 50, 100, 200, and 400 μg/mL) and 1 μg/mL of lipopolysaccharide (LPS) derived from *Escherichia coli* were added to the cells. After an additional 24 h of incubation, the culture supernatants were gently aspirated, and each well received 100 μL of fresh culture medium containing 10 μL of MTT solution. The plates were then incubated for 4 h at 37 °C, after which the absorbance was measured at 490 nm using a fluorescence microplate reader (SuPerMax, Shanghai, China). Cell viability relative to the untreated control group was calculated as a percentage.

#### 2.4.3. Evaluation of Phagocytic Uptake Capacity

The ability of RAW 264.7 macrophages to perform phagocytic uptake was assessed through the use of the neutral red assay [32]. RAW 264.7 cells (1 × 10^5^ cells/mL) were initially seeded in 96-well plates and allowed to incubate for 24 h. The cells were then treated with 100 μL of EPS-1 at concentrations of 0, 5, 6.25, 12.5, 25, 50, 100, 200, and 400 μg/mL, in addition to LPS at 1 μg/mL. Following treatment, 100 μL of neutral red was added to each well. After incubating for 2 h, the supernatant was discarded, and each well received 200 μL of cell lysis buffer, shaken gently for 60 min. Absorbance readings were taken at 540 nm using a fluorescence microplate reader (SuPerMax, China).

#### 2.4.4. Quantification of NO and Cytokines

To quantify nitric oxide (NO) and cytokine levels, RAW 264.7 macrophages were cultured in 6-well plates at a density of 1 × 10^5^ cells/mL and allowed to incubate for 24 h. The cells were then treated with varying concentrations of EPS-1 (0, 5, 6.25, 12.5, and 25 μg/mL) alongside LPS at a concentration of 1 μg/mL. After an additional 24 h incubation period, the supernatants were collected for analysis. NO levels were determined using nitric oxide assay kits (Beijing Boxbio Science & Technology Co., Ltd., Beijing, China), in accordance with established protocols. Furthermore, cytokines IL-6 and TNF-α were quantified using ELISA kits from Jiangsu Meimian Industrial Co., Ltd. (Yancheng, China), following the manufacturer’s instructions.

#### 2.4.5. Quantification of iNOS, IL-6, and TNF-α mRNA Levels via qRT-PCR

Total RNA was extracted from the RAW 264.7 cells using the QIAwave RNA Mini Kit (QIAGEN, GER, Hilden, Germany). The extracted RNA was reverse-transcribed using the PrimeScript Fast RT reagent kit with gDNA Eraser (Takara, Japan). The expression levels of iNOS, IL-6, and TNF-α genes were evaluated using the SsoAdvanced Universal SYBR Green Supermix (BioRad, Hercules, CA, USA) on the CFX96 Real-Time PCR System (Bio-Rad, USA). GAPDH served as the internal control. Using the 2^−ΔΔct^ method, the fold change in expression levels was quantified, and qRT-PCR experiments were executed in triplicate. The sequences of the primers are provided in Table A1.

#### 2.4.6. Western Blot

RAW 264.7 cells were treated with various concentrations of EPS-1 (0, 5, 6.25, 12.5, and 25 μg/mL) and LPS (1 μg/mL) for 24 h at 37 °C. Protein extraction was conducted using RIPA buffer in accordance with the manufacturer’s instructions and established methods. A 5–10% SDS-PAGE (Solarbio, Beijing, China) was employed to separate proteins, which were subsequently transferred to a 0.22 μm PVDF membrane (Millipore, Burlington, MA, USA). To prevent non-specific interactions, membranes were treated with 5% skim milk for 1 h and then incubated overnight at 4 °C with primary antibodies targeting p-p65, p65, p-IκBα, IκBα, p-ERK1/2, ERK1/2, and β-actin. HRP-conjugated secondary antibodies were applied for 1 h at room temperature. Protein expression was detected using the ECL Western blotting detection system (ChemiDoc™ XRS; Bio-Rad, Shanghai, China), and band intensities were quantified using ImageJ 2 software.

### 2.5. Statistical Analysis

The results were presented as means ± standard deviation (SD) from three independent replicates. Significant differences were assessed using Student’s *t*-test analysis and analysis of variance (ANOVA), followed by the Tukey–Kramer test in case of significant effect performed with GraphPad Prism 9.5.0 (GraphPad, Inc., San Diego, CA, USA). A *p* < 0.05 was considered statistically significant.

## 3. Results and Discussion

### 3.1. Extraction and Purification

**Figure 1:**
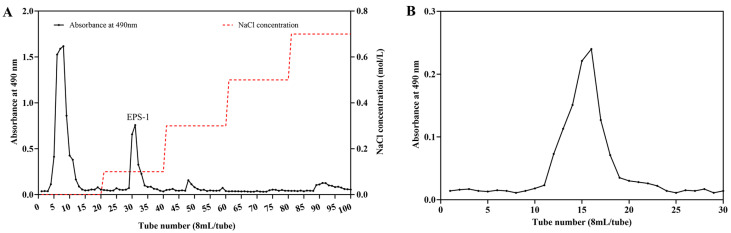
Chromatography of the polysaccharides from *A. jinshanensis* (EPS-1) by DEAE-Sepharose Fast Flow (**A**) and Sepharose CL-6B (**B**).

Crude EPS was extracted from *A. jinshanensis* BJ01 through a series of steps including deproteinization, ethanol precipitation, and lyophilization, resulting in a yield of 212.19 mg/L. The polysaccharides were initially fractionated using DEAE-Sepharose Fast Flow column chromatography. The fraction (EPS-1) was eluted using a 0.1 M NaCl solution (Figure 1A). Further purification was achieved through Sepharose CL-6B molecular sieve chromatography, which produced a well-defined symmetrical peak on the elution profile, as illustrated in Figure 1B. The obtained fraction was isolated, concentrated, dialyzed, and freeze-dried to prepare it for further characterization.

### 3.2. Characterization of EPS-1

#### 3.2.1. UV-vis Spectroscopy Analysis

**Figure 2:**
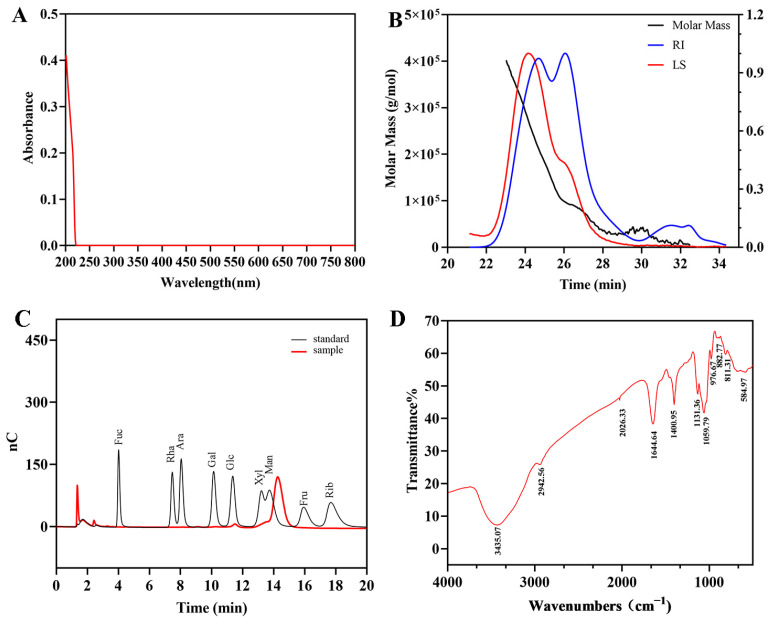
UV-vis spectrum (**A**), molecular weight analysis diagram (**B**), HPAEC chromatogram of standard monosaccharides and EPS-1 (**C**), and FT-IR spectrum (**D**) of EPS-1.

The chemical compositions of EPS-1 were summarized in Table 1. Ultraviolet and visible spectrum (UV-vis) scanning across the full wavelength range revealed no notable absorbance peaks at 260 nm and 280 nm (Figure 2A), indicating negligible amounts of protein or nucleic acids in EPS-1.

#### 3.2.2. Moisture Content, Solubility, Viscosity, and Gelling Capacity of EPS-1

Using a moisture analyzer, the water content of freeze-dried EPS-1 powder was determined to be 3.38%, indicating that the freeze-drying process was effective in removing moisture and ensuring the dryness and stability of the product. This result provides a solid foundation for subsequent storage and applications. Additionally, the solubility of EPS-1 was measured to be 78.67%, suggesting high solubility in water, which has potential advantages for applications in fields such as food and pharmaceuticals, where high water solubility of macromolecular materials is required.

In viscosity testing, the viscosity (η) of EPS-1 at a shear rate of 11 s^−1^ was measured to be 3.955 mPa·s, and within the shear rate range of 0–100 s^−1^, the viscosity remained constant, exhibiting the typical characteristics of a Newtonian fluid. This property indicates that the molecular structure of EPS-1 remains stable under shear forces, making it suitable for applications requiring fluid stability, such as emulsifiers or stabilizers in food systems.

Furthermore, the gel-forming ability of EPS-1 was evaluated using the inverted test tube method. The results showed that a 1% EPS-1 solution, after being treated at 95 °C and stored at 4 °C for 24 h, remained in a flowable state and failed to form a gel network. This indicates that EPS-1 lacks significant gel-forming capability, which may be related to its molecular aggregation behavior, branching structure, or the distribution of specific functional groups.

#### 3.2.3. Molecular Weight Determination, and Monosaccharide Analysis

The molecular weight of EPS-1 was determined by gel filtration chromatography using a series of Ohpak SB-805 HQ and SB-803 HQ columns (300 × 8 mm). The molecular weight of EPS-1 was calculated using the Mark–Houwink equation. Chromatographic data were processed with ASTRA 6.1 software. As shown in Figure 2B, the results include absolute molecular weight and molecular conformation analysis. The number-average molecular weight (Mn), weight-average molecular weight (Mw), and peak molecular weight (Mp) of EPS-1 were determined to be 69.65 kDa, 156.58 kDa, and 97.40 kDa, respectively. EPSs exceeding 10^5^ Da typically exhibit high viscosity and film-forming capacity, suggesting that EPS-1 may serve as a natural thickener in food systems or as a precursor for bio-based materials [6].

The monosaccharide composition of EPS-1 was analyzed using hydrolysis and ion-exchange chromatography. The results revealed that EPS-1 consists of mannose, xylose, and glucose, with a molar ratio of 30.38:5.78:1.00 (Figure 2C), mannose was identified as the predominant monosaccharide of EPS-1. It is worth noting that a slight retention time shift was observed for the mannose peak when compared to the standard. This discrepancy can be attributed to common instrumental factors in HPAEC, where retention time variations of 0.1–0.3 min are frequently observed under standard operating conditions. Such shifts may result from subtle fluctuations in column temperature, changes in injection order, or matrix effects from sample hydrolysates. Mannose is known to interact with mannose-specific lectin receptors on immune cells, enhancing immune responses [33]. Moreover, guar gum and konjac gum, both classified as mannose-based polysaccharides, serve not only as natural thickeners and gelling agents to maintain food structure and texture but also as dietary fibers and prebiotics that contribute to nutritional regulation and gut health. These characteristics suggest that EPS-1 may have promising applications in the food industry [34].

#### 3.2.4. FT-IR Spectroscopy

FT-IR spectroscopy was employed to analyze the primary functional groups and chemical bonds of EPS-1. The spectral region between 3600 and 3200 cm^−1^ shown a characteristic O-H stretching vibration associated with hydroxyl (-OH) groups. Notably, the peak at 3435.07 cm^−1^ indicates strong O-H stretching, characteristic of polysaccharides with hydroxyl-rich backbones (Figure 2D). This structural feature promotes hydrogen bonding, which was essential for the bioactivities often associated with polysaccharides, such as antioxidant and immunomodulatory effects [35]. The absorption peak at 2942.56 cm^−1^ corresponds to C-H stretching vibrations, indicating the presence of aliphatic chains commonly found in polysaccharide structures. Additionally, the peak at 1059.79 cm^−1^ indicates C-O stretching vibration, supporting the presence of ether linkages and highlighting the glycosidic bonds in the polysaccharide. These bonds, commonly found in polysaccharides, play a key role in structural stability and biological activity by forming the backbone [36].

#### 3.2.5. Methylation Analysis

**Figure A1:**
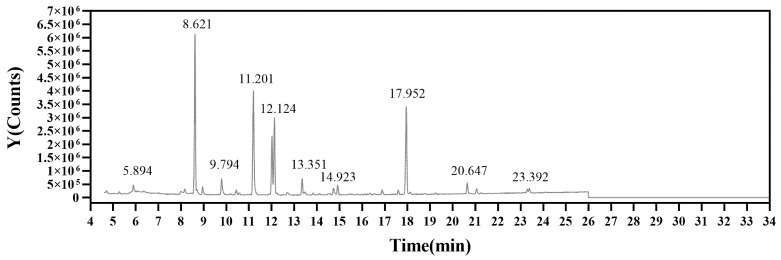
Total ion current chromatogram of EPS-1.

To analyze the glycosidic linkages in EPS-1, partially methylated alditol acetates (PMAAs) were produced via methylation, hydrolysis, and acetylation. The resulting PMAAs were then analyzed via GC-MS, enabling a detailed characterization of the polysaccharide’s structural components. This method is particularly effective for elucidating glycosidic linkages, as it allows for precise identification of sugar units and linkage types, which are critical for understanding the bioactivity of EPS-1 [37]. The distinct peaks identified in Figure A1 correspond to specific structural features and compositional attributes of the methylated polysaccharides, as inferred from their retention times and relative intensities. The peak at 5.89 min suggests a low-molecular-weight, highly volatile methylated product, such as a monosaccharide or a simple sugar structure derivative. Peaks at 8.62 and 9.79 min, with medium retention times, may indicate complex sugar units or partially methylated polysaccharide structures. Peaks at 11.20, 12.12, and 13.35 min suggest higher molecular weights or complex structures, likely representing fully methylated polysaccharide branches. The peaks at 14.92, 17.95, 20.64, and 23.39 min were typically associated with highly methylated, structurally complex polysaccharides, potentially containing multiple sugar residues and branching structures. The relative molar ratios of various glycosidic bonds were estimated by calculating the ratio of chromatographic peak areas to the corresponding derivative molecular weights, as shown in Table 2. This analysis facilitates a detailed hypothesis regarding the structural features of EPS-1, encompassing the degree of branching, the specific sugar residues present, and the types of glycosidic linkages involved. To further elucidate these results, the specific sugar units and their linkages will need to be determined through NMR spectroscopy.

#### 3.2.6. NMR Spectroscopy Analysis

This study integrated both 1D-NMR and 2D-NMR techniques, including COSY and HSQC, HMBC, and NOESY, to systematically characterize the chemical shifts of hydrogen and carbon atoms in each sugar residue, thereby facilitating the elucidation of their inter-residue linkages [38,39].

**Figure 3:**
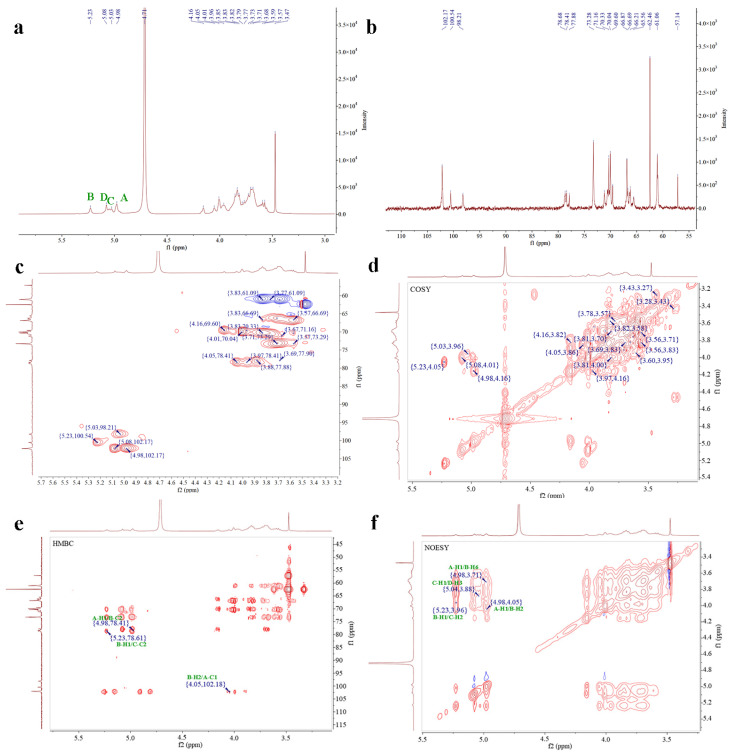
^1^H-NMR (**a**), ^13^C-NMR (**b**), HSQC (**c**), COSY (**d**), HMBC (**e**), and NOESY spectra (**f**) of EPS-1. The green A, B, C, and D in the image represent the four main sugar residues of EPS-1.

As shown in Figure 3, the ^1^H NMR spectrum of EPS-1 exhibited signals primarily within the range of 3.0–5.5 ppm. In the anomeric region (4.8–5.5 ppm), multiple coupling peaks were observed, indicating the presence of several α-configured sugar residues with chemical shifts at 4.98, 5.03, 5.08, and 5.23 ppm. The region from 3.1 to 4.2 ppm contained non-anomeric proton signals, with a strong peak at approximately 4.71 ppm corresponding to the solvent, and a signal near 3.47 ppm attributable to the hydrogen atoms of an O-CH_3_ group [7]. Based on the ^13^C NMR spectrum and cross-peaks observed in the HSQC spectrum, the chemical shifts at 4.98/102.17, 5.23/100.54, 5.03/98.21, and 5.08/102.17 ppm were assigned to sugar residues A, B, C, and D, respectively.

For residue A, COSY cross-peaks at 4.98/4.16, 4.16/3.82, 3.82/3.96, 3.96/3.70, 3.71/3.83, and 3.77 ppm allowed for the sequential assignment of H2–H6 with corresponding shifts of 4.16, 3.82, 3.96, 3.71, 3.83, and 3.77 ppm. HSQC data showed the ^13^C chemical shifts of C1–C6 as 102.17,69.60, 70.33, 69.59, 73.29, and 61.08 ppm. The downfield shift of C1 indicated a substitution at the O-1 position. Combined with methylation analysis and literature references, residue A was identified as α-D-Man*p*-(1→.

For residue B, COSY cross-peaks at 5.23/4.05, 4.05/3.86, 3.86/3.91, 3.91/3.61, 3.61/3.69, and 3.58 ppm supported the assignment of H2–H6, with chemical shifts of 4.05, 3.86, 3.91, 3.61, and 3.69/3.58 ppm. The corresponding ^13^C shifts (C1–C6) were 100.54, 78.41, 70.30, 69.72, 73.39, and 66.88 ppm, with downfield shifts of C1, C2, and C6 suggesting substitutions at O-1, O-2, and O-6. Therefore, residue B was identified as →2,6)-α-D-Man*p*-(1→.

Residue C showed COSY cross-peaks at 5.03/3.96, 3.96/3.68, 3.68/3.85, 3.85/3.57, 3.57/3.69, and 3.81 ppm, corresponding to H2–H6 with shifts of 3.96, 3.68, 3.85, 3.57, and 3.69/3.81 ppm. HSQC analysis indicated 13C chemical shifts of 98.21, 78.64, 71.17, 69.54, 70.34, and 61.04 ppm. The downfield shifts of C1 and C2 suggested substitutions at the O-1 and O-2 positions, and residue C was thus assigned as →2)-α-D-Man*p*-(1→.

Residue D exhibited COSY cross-peaks at 5.08/4.01, 4.01/3.88, 3.88/3.79, 3.79/3.76, and 3.76/3.61 ppm, indicating H2–H6 shifts of 4.01, 3.88, 3.79, 3.76, and 3.61 ppm. The HSQC spectrum revealed 13C shifts of 102.17, 70.04, 77.88, 69.60, 70.51, and 60.80 ppm for C1–C6. The downfield shifts of C1 and C3 suggested substitutions at the O-1 and O-3 positions, and residue D was determined to be →3)-α-D-Man*p*-(1→ [40]. The corresponding assignments are summarized in Table 3.

HMBC spectral analysis revealed potential linkage patterns among the sugar residues. The analysis revealed cross-peaks indicative of potential linkages among the sugar residues. Notably, the correlation between H1 of residue A and C2 of residue B, observed at 4.98/78.41 ppm, alongside the signal between C1 of A and H2 of B at 102.18/4.05 ppm, supports the presence of a linkage between residues A and B. Similarly, the cross-peak between H1 of residue B and C2 of residue C at 5.23/78.64 ppm suggests a potential linkage between residues B and C. Furthermore, the NOESY spectrum reinforced these observations, highlighted by the cross-peak at 4.98/4.05 ppm between H1 of residue A and H2 of B. Additional cross-peaks were noted, including H1 of sugar residue A with H6 of B at 4.98/3.69 ppm, H1 of B with H2 of C at 5.23/3.96 ppm, and H1 of C with H3 of D at 5.03/3.88 ppm.

**Figure 4:**
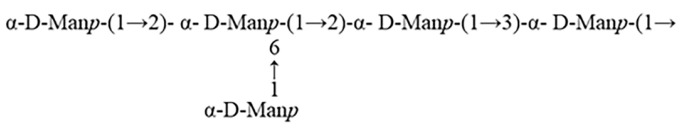
The major repeating unit of EPS-1.

In summary, the linkages consisted of EPS-1 mainly include α-D-Man*p*-(1→, →2,6)-α-D-Man*p*-(1→, →2)-α-D-Man*p*-(1→, and →3)-α-D-Man*p*-(1→. Methylation analysis detected small amounts of xylose and glucose in EPS-1 that were not clearly identified by NMR. The discrepancies between the characterization results may be attributed to the following reason. Methylation analysis and NMR employ different analytical principles; methylation analysis using GC-MS offers higher detection sensitivity for specific linkage types, while NMR primarily relies on sufficient signal intensity for accurate characterization [8]. Notably, according to the methylation analysis results (Table 2), the molar ratios of 2-Xyl*p* and glucose-containing linkage types such as 3,4-Glc*p* and 2,3,6-Glc*p* were only 2.19%, 2.11%, and 1.17%, respectively, confirming the minor nature of these components in the polysaccharide structure. Therefore, based on the predominance of mannose, the structural characterization of EPS-1 was focused on the linkage patterns and spatial arrangement of mannose residues. This strategy aligns with the common practice in polysaccharide structural characterization of prioritizing the analysis of the main structural components [41,42]. Drawing upon prior studies on mannose-rich polysaccharides, EPS-1 is likely to possess potential biological activities such as antioxidant, immunomodulatory, and gut microbiota-regulating properties [43]. Based on the analytical data, EPS-1 primarily consisted of chains composed of →2,6)-α-D-Man*p*-(1→, →2)-α-D-Man*p*-(1→, and →3)-α-D-Man*p*-(1→ units. The side chains are made up of α-D-Man*p*-(1→ units linked to the O-2 and O-6 positions of the →2,6)-α-D-Manp-(1→ residues. The major repeating unit of EPS-1 is proposed as shown in Figure 4. The molecular weight of this repeating unit is 648.46 Da, calculated based on the molecular weight of mannose (180.16 Da) and accounting for the loss of water during glycosidic bond formation. By dividing the Mn value (69.653 kDa) by the molecular weight of the repeating unit (648.46 Da), the degree of polymerization (DP) is estimated to be approximately 107. This structural information provides critical insights into the nature of EPS-1. The fact that EPS-1 is primarily composed of mannose may be a key factor contributing to its lack of gelling ability, similar to konjac glucomannan, which exhibits gelling properties after alkaline treatment to remove acetyl groups [44].

#### 3.2.7. SEM and AFM Observation of EPS-1

**Figure 5:**
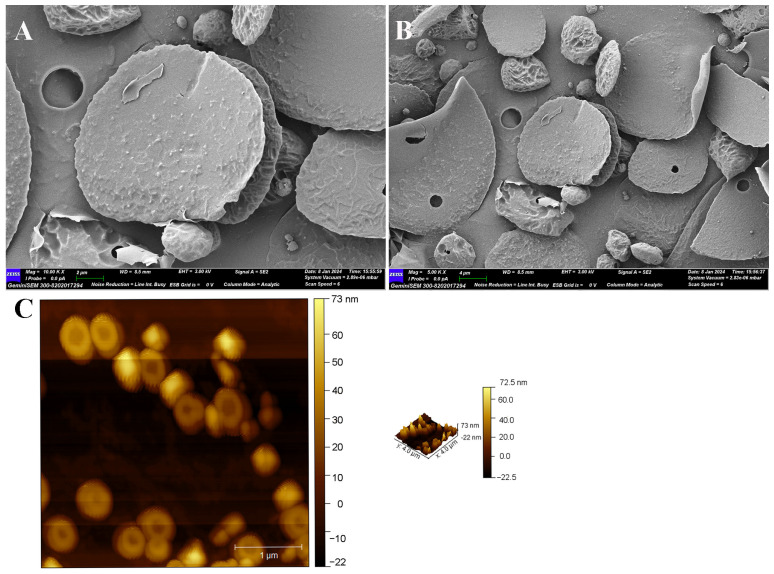
SEM (**A**) magnification 10 K×, scale bar 2 μm, (**B**) magnification 5 K×, scale bar 4 μm, and AFM (**C**) images of EPS-1.

The morphology characteristic of EPS-1 was analyzed using SEM. As shown in Figure 5A,B, at a magnification of 10 K and 5 K, EPS-1 predominantly exhibited a round, flake-like morphology, with a smooth surface. The surface of EPS-1 was characterized by spherical formations, distinguishing it from other EPSs, such as those produced by *Lactobacillus pantheris* TCP102 [45] and other flakes structures [46]. The smooth surface of EPS-1 indicates a relatively uniform molecular arrangement, which may enhance its water-holding capacity [47]. Additionally, the irregular edges of the particles may result from shrinkage during drying or from EPS-1 self-assembly during production [48].

AFM, a widely used technique for examining the morphology, shape, and aggregation characteristics of polysaccharides, was employed to further investigate EPS-1. AFM analysis was performed at a concentration of 10 μg/mL, and the results are shown in Figure 5C. EPS-1 exhibited a predominantly spherical morphology with relatively small particle sizes and moderately aggregated particles with a maximum height of 72.5 nm. The particle size may explain the system’s Newtonian fluid behavior, consistent with the viscosity results. Although slight aggregation was observed among some particles, the overall distribution was uniform, indicating good homogeneity and dispersibility. This moderate aggregation may help explain the observed solubility results, as polysaccharides with smaller and less aggregated particles tend to exhibit better solubility under similar molecular weight conditions.

### 3.3. Immunomodulatory Activity of EPS-1

#### 3.3.1. Impact of EPS-1 on RAW 264.7 Cell Viability

**Figure 6:**
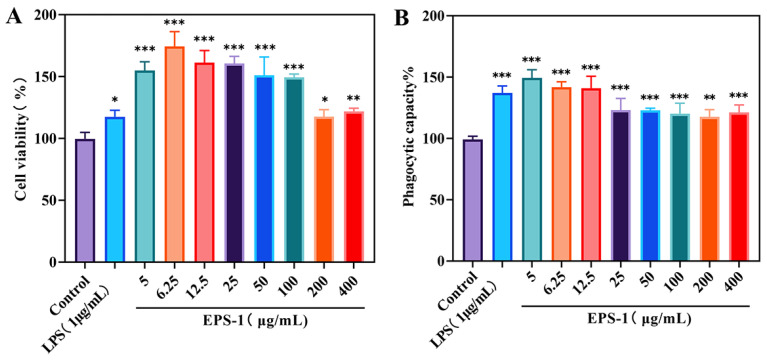
Effect of different concentrations (0–400 μg/mL) of EPS-1 treatment for 24 h on cell viability (**A**) and phagocytic capacity (**B**). Data are shown as means ± SD of three independent experiments. Compared with the control, * *p* < 0.05, ** *p* < 0.01, and *** *p* < 0.001.

Before assessing the immunomodulatory effects of EPS-1, we evaluated its cytotoxic potential on RAW 264.7 cells utilizing the MTT assay. Cell proliferation was measured after treating RAW 264.7 cells with a range of EPS-1 concentrations (5, 6.25, 12.5, 25, 50, 100, 200, and 400 μg/mL) for 24 h. As shown in Figure 6A, EPS-1 significantly enhanced cell proliferation across all tested concentrations (*p* < 0.05), with the most pronounced increase observed at 6.25 μg/mL. These findings suggest that EPS-1 effectively stimulates RAW 264.7 cell activation. This observation aligns with previous research demonstrating that polysaccharides from microbial sources can enhance immune responses in RAW 264.7 cells [49,50].

#### 3.3.2. Influence of EPS-1 on Phagocytic Activity in RAW 264.7 Cells

The effects of EPS-1 on the phagocytic activity of RAW 264.7 cells were assessed using neutral red, an acid–base indicator that stains viable cells. Upon cellular uptake, neutral red accumulates in lysosomes, forming red deposits. As shown in Figure 6B, treatment with varying concentrations of EPS-1 (5, 6.25, 12.5, 25, 50, 100, 200, and 400 μg/mL) for 24 h significantly enhanced the phagocytic activity of RAW 264.7 cells compared to the blank control group (*p* < 0.05). At a concentration of 5 μg/mL, the absorbance (OD_540_) reached 0.6702, representing a 49.2% increase over the control, thereby confirming that EPS-1 substantially enhances the phagocytic capacity of RAW 264.7 cells. The results provide valuable insights for future immunological assessments; similar results have been observed with other LAB-derived EPS, where macrophage activation through enhanced phagocytic activity was identified as a key immunomodulatory mechanism [51,52].

#### 3.3.3. Influence of EPS-1 on the Production of NO, IL-6, and TNF-α in RAW 264.7 Cells

The activation of macrophages triggers an increase in iNOS activity, which promotes the transformation of L-arginine and molecular oxygen into considerable amounts of NO [53]. NO serves as a vital intracellular signaling molecule, playing a key role in the defense against microorganisms and tumor cells. Cytokines are signaling proteins produced by immune cells in response to mitogen stimulation. They support the expansion and functional maturation of designated target cells, increase surface receptor expression, amplify anti-infective mechanisms, and fine-tune both immune responses and inflammatory processes [54]. IL-6 acts as a dual-function molecule, serving as both a growth factor for various cells and a key player in immune differentiation. It promotes the proliferation of T and B lymphocytes, augments antibody synthesis in B cells, and facilitates hematopoiesis [55]. TNF-α serves as a critical immunomodulatory cytokine that facilitates T cell proliferation and differentiation, thereby enhancing their cytotoxic functions. Additionally, it stimulates B cell proliferation and augments the cytotoxic capabilities of various immune cell populations [56].

**Figure 7:**
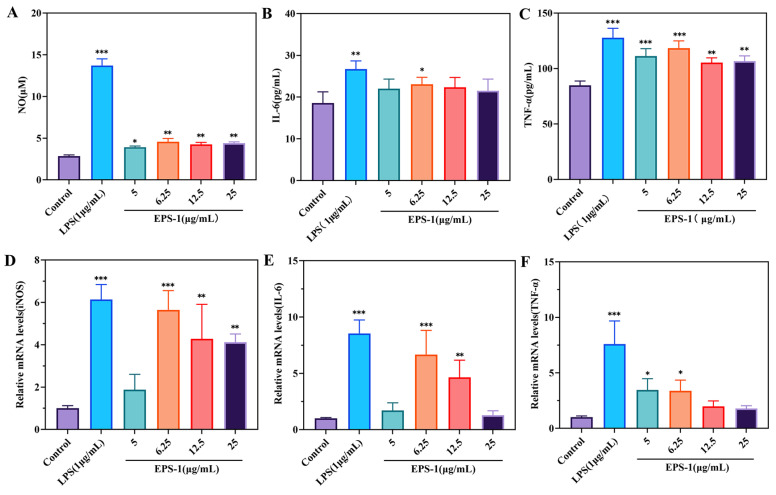
The cytokines included NO (**A**), IL-6 (**B**), TNF-α (**C**), and their mRNA expression levels (**D**–**F**) in RAW 264.7 cells treated with different concentrations (0–25 μg/mL) of EPS-1. Data are shown as means ± SD of three independent experiments. Compared with the control, * *p* < 0.05, ** *p* < 0.01, and *** *p* < 0.001.

As shown in Figure 7A, EPS-1 enhanced NO production in macrophages compared to the control group, with the highest level observed at a concentration of 6.25 μg/mL. This finding is consistent with previous studies showing that exopolysaccharides from lactic acid bacteria can stimulate the release of NO, enhance the phagocytic activity of macrophages, and thereby improve their immunomodulatory function [57]. In addition, EPS-1 was able to promote the release of IL-6 and TNF-α from RAW 264.7 cells. At a concentration of 6.25 μg/mL, the IL-6 level increased to 23.078 pg/mL, slightly lower than that induced by LPS treatment (26.708 pg/mL) (Figure 7B). Furthermore, the TNF-α concentration increased significantly, reaching 118.363 pg/mL at 6.25 μg/mL, just below the 127.763 pg/mL induced by LPS (Figure 7C). These cytokines were critical mediators of the immune response, facilitating the recruitment and activation of additional immune cells. The activation of these cytokines through pathways such as NF-κB and MAPK has been widely observed in studies on polysaccharides from LAB and other sources, which trigger macrophage activation through these signaling cascade [58].

#### 3.3.4. Impact of EPS-1 on mRNA Levels of iNOS, IL-6, and TNF-α

qRT-PCR analysis was conducted to assess the impact of EPS-1 on mRNA expression levels of iNOS, IL-6, and TNF-α in the RAW 264.7 cell line. The data depicted in Figure 6 indicate that EPS-1 substantially enhanced the mRNA levels of these cytokines compared to untreated controls. In the group treated with 6.25 μg/mL of EPS-1, iNOS expression increased by 5.61 times compared to the control group (Figure 7D). Similarly, IL-6 levels were elevated by 6.66 times in the EPS-1 treated group at the same concentration (Figure 7E). Additionally, TNF-α expression increased by 3.44 times in the group treated with 5 μg/mL of EPS-1 (Figure 7F). In summary, EPS-1 significantly upregulated the mRNA expression levels of iNOS, IL-6, and TNF-α in RAW 264.7 cells, indicating its strong immunomodulatory potential. These findings further confirm the bioactivity of EPS-1 in promoting the expression of immune-related cytokines.

#### 3.3.5. Western Blot

NF-κB transcription factors are pivotal regulators of gene expression, modulating a broad spectrum of genes involved in critical cellular and physiological processes. These include the orchestration of immune responses, the modulation of inflammatory pathways, cell adhesion, differentiation, oxidative stress responses, and apoptosis. The complexity of NF-κB signaling underlies its involvement in both innate and adaptive immunity, as well as its role in maintaining cellular homeostasis under stress conditions [59]. The NF-κB family in the immune system comprises five key proteins: p65, RelB, c-Rel, p50, and p52 [60]. The MAPK pathway, encompassing the p38, ERK, and JNK subtypes, is essential for mediating macrophage immune functions and regulating the transcription of iNOS and COX-2 [18].

**Figure 8:**
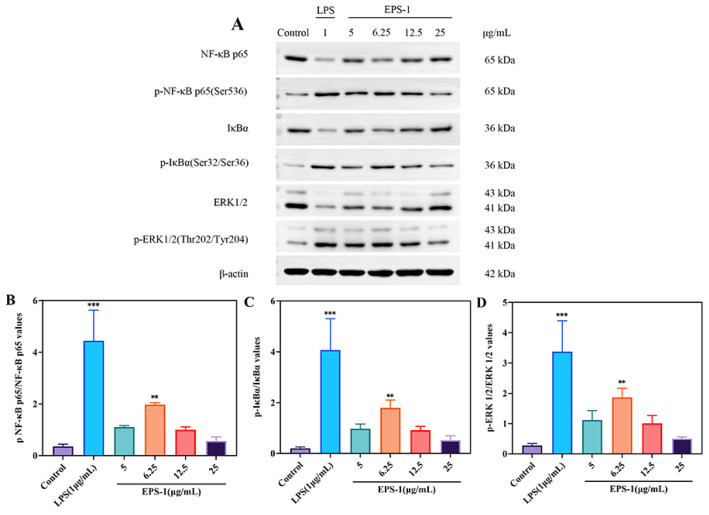
Effect of EPS-1 on the NF-κB and MAPK signaling pathway in RAW 264.7 cells. The expression of proteins in RAW 264.7 cells was assessed by Western blotting (**A**–**D**). β-actin served as a normalization control. The data are presented as the mean ± SD of three independent experiments. ** *p* < 0.01, and *** *p* < 0.001 compared with the control group.

In vitro studies assessing immunoactivity measured the levels of signaling-pathway-related proteins p65, IκBα, and ERK. As shown in Figure 8, treatment of RAW 264.7 cells with 6.25 μg/mL EPS-1 significantly increased the expression of phosphorylated p65 (p-p65), phosphorylated IκBα (p-IκBα), and phosphorylated ERK (p-ERK) proteins compared to the untreated control group (** *p* < 0.01). This observation aligns with previous findings demonstrating that mannose-rich microbial polysaccharides can regulate the NF-κB/MAPK signaling pathway, thereby promoting cytokine expression [61,62,63]. EPS-1 has the potential to act as an immune stimulant by enhancing macrophage activity. Its role in modulating immune responses and reducing inflammation is promising and deserves further study to fully understand its mechanisms and applications.

## 4. Conclusions

In this study, a novel exopolysaccharide (EPS) was successfully isolated and characterized from *Acetilactobacillus jinshanensis* BJ01, a strain derived from traditional Chinese *Baijiu* fermentation mash. The comprehensive structural analysis revealed that the purified EPS-1 is primarily composed of mannose, xylose, and glucose, with a molar ratio of 30.3:5.78:1.00, and exhibits a molecular weight of 156.58 kDa. Methylation analysis and NMR spectroscopy further clarified the types of constituent sugar residues and glycosidic linkages, indicating that EPS-1 is primarily composed of →α-D-Man*p*-(1→, →2,6)-α-D-Man*p*-(1→, →2)-α-D-Man*p*-(1→, and →3)-α-D-Man*p*-(1→. In vitro assays demonstrated that EPS-1 significantly activated RAW 264.7 macrophages, enhancing NO production and promoting the secretion of pro-inflammatory cytokines such as IL-6 and TNF-α. Moreover, Western blot analysis confirmed that EPS-1 activated the NF-κB and MAPK signaling pathways, supporting its role in modulating immune responses. These results not only validate the immunostimulatory potential of EPS-1 but also highlight its mechanism of action via key inflammatory pathways. Collectively, the findings suggest that EPS-1 may serve as a promising candidate for use as an immunomodulatory agent or as a bioactive ingredient in functional food applications.

## Figures and Tables

**Table 1:** Molecular weight and relative monosaccharide composition of EPS-1.

Parameters	EPS-1
Mn(kDa)	69.65
Mw	156.58
Mz	220.03
Monosaccharides	(mg/g)
Mannose	391.49
Xylose	62.11
Glucose	12.88

**Table 2:** Results of the methylation analysis for EPS-1.

Linkage Type	Mass Fragments (*m/z*)	Molar Ratio (%)
t-Man*p*	87, 102, 118, 129, 145, 161, 162, 205	33.80
2-Xyl*p*	88, 101, 117, 129, 130, 161, 190	2.19
3-Man*p*	87, 101, 118, 129, 161, 202, 234	14.72
2-Man*p*	88, 101, 129, 130, 161, 190, 205	19.44
6-Man*p*	87, 99, 102, 118, 129, 162, 189, 233	4.09
3,4-Glc*p*	87, 118, 129, 143, 185, 203, 305	2.11
2,6-Man*p*	87, 88, 99, 100, 129, 130, 189, 190	22.48
2,3,6-Glc*p*	71, 87, 100, 129, 130, 159, 160, 189, 202, 262	1.17

Note: Relative molar amount = peak area/molecular weight; relative molar ratio (%) = relative molar amount/sum of relative molar amounts of all components.

**Table 3:** Chemical shift data for the glycosyl residues of EPS-1.

Code	Residues	Chemical Shifts (δ, ppm)					
		H1/C1	H2/C2	H3/C3	H4/C4	H5/C5	H6,6′/C6
A	α-D-Man*p*-(1→	4.98/102.17	4.16/69.60	3.82/70.33	3.96/69.59	3.71/73.29	3.83,3.77/61.08
B	→2,6)-α-D-Man*p*-(1→	5.23/100.54	4.05/78.41	3.86/70.30	3.91/69.72	3.61/73.39	3.69,3.58/66.88
C	→2)-α-D-Man*p*-(1→	5.03/98.21	3.96/78.64	3.68/71.17	3.85/69.54	3.57/70.34	3.69,3.81/61.04
D	→3)-α-D-Man*p*-(1→	5.08/102.17	4.01/70.04	3.88/77.88	3.79/69.60	3.76/70.51	3.61/60.80

## Data Availability

The original contributions presented in the study are included in the article, further inquiries can be directed to the corresponding authors.

## Appendix A

**Table A1:** The primer sequences.

Primer	Primer Sequences
iNOS	Forward (5′-3′): GACGAGACGGATAGGCAGAGATTG
	Reverse (5′-3′): AACTCTTCAAGCACCTCCAGGAAC
IL-6	Forward (5′-3′): TCTATACCACTTCACAAGTCGGA
	Reverse (5′-3′): GAATTGCCATTGCACAACTCTTT
TNF-α	Forward (5′-3′): ACGCTCTTCTGTCTACTGAACTTCG
	Reverse (5′-3′): TGGTTTGTGAGTGTGAGGGTCTG
GAPDH	Forward (5′-3′): GCAAATTCAACGGCACAGTCAAG
	Reverse (5′-3′): TCGCTCCTGGAAGATGGTGATG
